# The Journey from Traffic Offender to Severe Road Trauma Victim: Destiny or Preventive Opportunity?

**DOI:** 10.1371/journal.pone.0122652

**Published:** 2015-04-22

**Authors:** Kwok M. Ho, Sudhakar Rao, Maxine Burrell, Tarun S. Weeramanthri

**Affiliations:** 1 Department of Intensive Care Medicine, Royal Perth Hospital, Perth, Australia; 2 School of Population Health, University of Western Australia, Perth, Australia; 3 Murdoch University, Perth, Australia; 4 State Trauma Unit, Royal Perth Hospital, Perth, Australia; 5 Public Health and Clinical Services Division, Western Australia Department of Health, Perth, Australia; Örebro University, SWEDEN

## Abstract

**Background:**

Road trauma is a leading cause of death and injury in young people. Traffic offences are common, but their importance as a risk indicator for subsequent road trauma is unknown. This cohort study assessed whether severe road trauma could be predicted by a history of prior traffic offences.

**Methodology and Principal Findings:**

Clinical data of all adult road trauma patients admitted to the Western Australia (WA) State Trauma Centre between 1998 and 2013 were linked to traffic offences records at the WA Department of Transport. The primary outcomes were alcohol exposure prior to road trauma, severe trauma (defined by Injury Severity Score >15), and intensive care admission (ICU) or death, analyzed by logistic regression. Traffic offences directly leading to the road trauma admissions were excluded. Of the 10,330 patients included (median age 34 years-old, 78% male), 1955 (18.9%) had alcohol-exposure before road trauma, 2415 (23.4%) had severe trauma, 1360 (13.2%) required ICU admission, and 267 (2.6%) died. Prior traffic offences were recorded in 6269 (60.7%) patients. The number of prior traffic offences was significantly associated with alcohol-related road trauma (odds ratio [OR] per offence 1.03, 95% confidence interval [CI] 1.02–1.05), severe trauma (OR 1.13, 95%CI 1.14–1.15), and ICU admission or death (OR 1.10, 95%CI 1.08–1.11). Drink-drinking, seat-belt, and use of handheld electronic device offences were specific offences strongly associated with road trauma leading to ICU admission or death—all in a ‘dose-related’ fashion. For those who recovered from road trauma after an ICU admission, there was a significant reduction in subsequent traffic offences (mean difference 1.8, 95%CI 1.5 to 2.0) and demerit points (mean difference 7.0, 95%CI 6.5 to 7.6) compared to before the trauma event.

**Significance:**

Previous traffic offences were a significant risk factor for alcohol-related road trauma and severe road trauma leading to ICU admission or death.

## Introduction

Death and injury of young people due to road trauma is a worldwide problem affecting both developing and developed countries [1]. According to the World Health Organisation’s *Global Status Report on Road Safety* [2], more than 1·2 million people die and up to 50 million others are injured on the world’s roads every year. The sad reality is that many of those who die or are injured with chronic disability after road trauma are young members of our society [1]. This is likely due to the fact that young road users do have a higher tendency to pursue risk-taking behaviours [3].

Multiple measures have been used to reduce the burden of road trauma, including improvement in road design, separation of motorised vehicles from pedestrians and cyclists [1], use of community or school-based education program [3], designated driver programs, and use of traffic laws to enforce road-safety [4]. In many developed countries, traffic laws including compulsory wearing of seat-belts or helmets, safe speed limits, and driving without the influence of alcohol or drugs are common. Most traffic law systems involve a range of penalties, including monetary fines, demerit or penalty points and, for severe or repeated offences, suspension of the driver’s licence. Early evidence suggested that the initiation of traffic laws involving demerit or penalty points dramatically reduced total number of fatalities on the roads in some countries [4,5]. However, this benefit was not sustained [4,6].

Traffic offences are common in many developed countries [4–7], but their importance as a risk indicator for subsequent road trauma is unknown. We hypothesized that prior traffic offences, especially if they are repeated or severe, are an important risk factor for subsequent severe road trauma leading to intensive care unit (ICU) admission or death compared to those without prior traffic offences. In this cohort study, we aimed to assess whether severe road trauma could be predicted by a history of prior traffic offences.

## Methods

### Study design

This study was approved by Royal Perth Hospital Ethics Committee (EC13-004) and Western Australia (WA) Department of Transport (A3106769). Consent was waived by the Ethics Committee due to observational nature of the study and only de-identified data were used in the final analysis and reporting. Clinical data of all adult road trauma patients admitted to the State Trauma Centre between 1998 and 2013 were linked to traffic offences records at the Department of Transport in this cohort study. The timing, nature, and the associated demerit points of all traffic offences of each patient were retrieved, including a separation of whether these occurred before and after their first index admission to the State Trauma Centre due to road trauma. Offences directly leading to the first index road trauma admissions were excluded.

The primary outcomes of this study were (i) alcohol exposure within 12 hours prior to road trauma, (ii) severe trauma (defined by an Injury Severity Score [ISS] >15), and (iii) intensive care admission (ICU) or death. The secondary outcomes were readmission due to road trauma and incidence of subsequent traffic offences after the first road trauma during the study period. The relationships between the primary outcomes and cumulative number of prior traffic offences (as an exposure) were analyzed, while adjusting for known risk factors for road trauma such as age, gender, and duration of having a driver’s licence. Because each traffic offence can be graded in its severity by demerit points, and the nature of some traffic offences could also be different to the others (e.g. bicycle offences vs. drink-driving), the significance of the cumulative demerit points and cumulative number of specific types of traffic offences, respectively, were also assessed. In assessing the relationships between traffic offences and the primary outcomes, we only included road trauma patients if they were drivers or motorcycle riders in the road traffic accidents.

As for the secondary outcomes, we also used logistic regression to assess whether traffic offences *subsequent* to the first road trauma admission were related to the risk of readmissions from subsequent road trauma, after adjusting for age, gender, and duration of having a driver’s licence. Except for those who died in their first trauma admission, all patients with a driver’s licence were included in this analysis, including those who were not drivers or motorcycle riders in the first index road trauma admission. When we assessed the association between severe road trauma admission and incidence of subsequent traffic offences, we conducted a sensitivity analysis by restricting the analysis to patients who had at least one traffic offence after their first road trauma to exclude any possible effect of disability, incurred in their first road trauma admission, in reducing subsequent traffic offences.

### Statistical analysis

We estimated that ICU admissions or fatalities occurred in about 5–10% of the total 10,000 road trauma admissions to the State Trauma Centre during the study period. We assumed that half of those motor vehicle drivers or motorcycle riders would have traffic offences prior to their road trauma, and prior traffic offence was associated with a relative risk of 2 or more in leading to ICU admission or death after road trauma. The study sample size (~10,000) would have >95% power to detect a statistical difference between those with and without prior traffic offences in their risk of requiring ICU admission or death after the road trauma.

We analysed the differences in categorical variables using Chi-squared tests and continuous variables with skewed distributions using Mann-Whitney tests. We used logistic regression models to assess the associations between traffic offences and primary or secondary outcomes. We modelled the primary outcomes and risk of readmission due to road trauma after the first road trauma admission as a dichotomised outcome in a logistic regression. During the modelling process, no variable was removed. In a sensitivity analysis for the primary outcome, we also assessed whether the cumulative number of demerit points was associated with the risk of subsequent severe trauma requiring ICU admission in a non-linear fashion, using a 4-knot restricted cubic spline function [8].

In this study, we considered a p value of <0·05 as statistically significant and used SPSS for Windows (version 22, 2014, IBM, IL, USA) and S-Plus (version 8.0, 2007. Insightful Corp., Seattle, Washington, USA) for all statistical analyses. The STROBE checklist can be accessed in the **S1 Text** and the full dataset can be accessed at http://dx.doi.org/10.5061/dryad.2k422.

## Results

**Fig 1:**
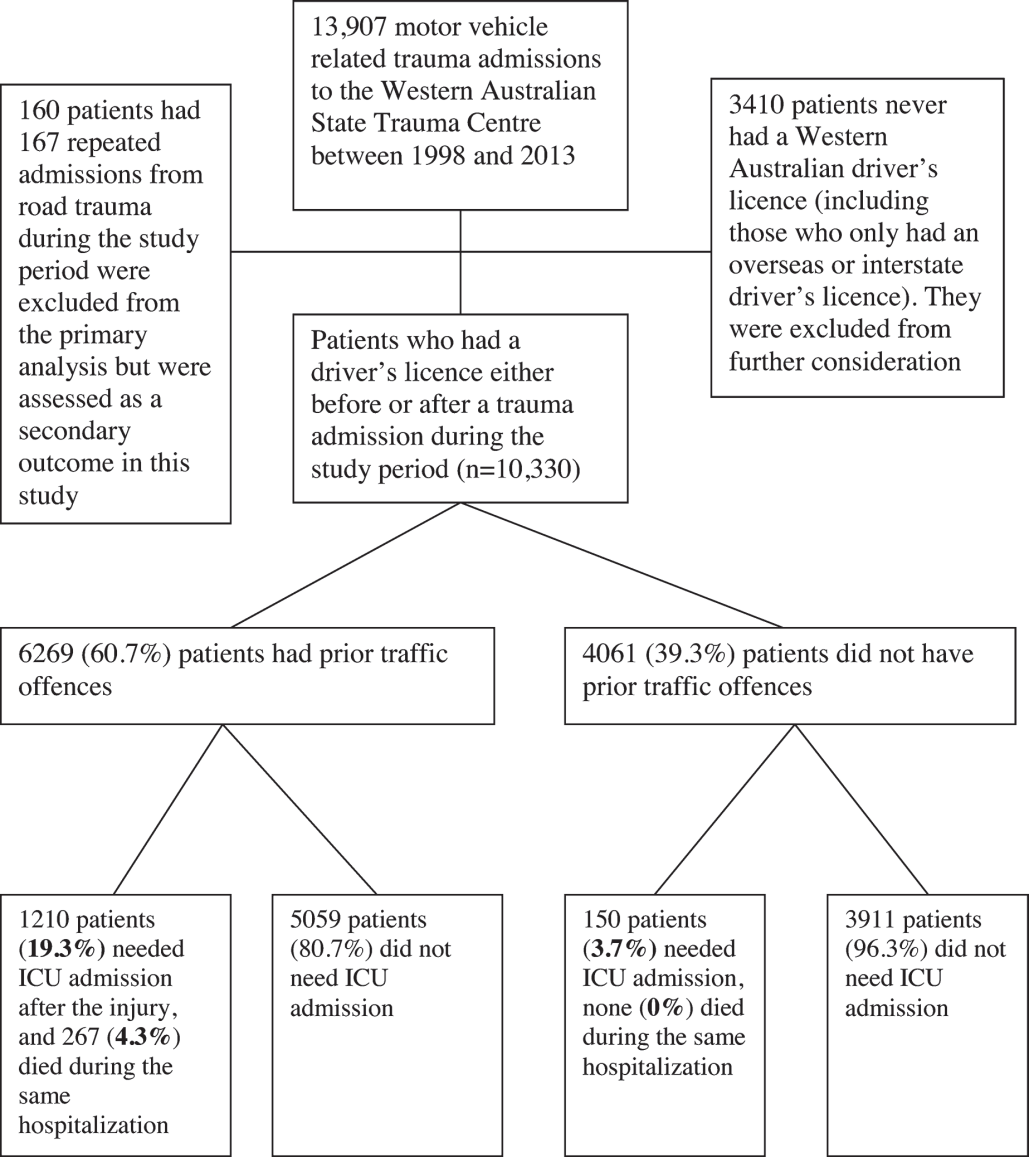
Flow chart showing inclusion and exclusion of patients for the study. ICU, intensive care unit.

Between January 1, 1998, and December 31, 2013, 13,907 episodes of road trauma admission to the State Trauma Centre were recorded; after excluding 3410 injured patients (mostly pedestrians or motor vehicle passengers) who never had a driver’s licence, and 160 patients who had 167 repeated admissions from road trauma injury during the study period, 10,330 patients were used in our analysis of the primary outcomes (**Fig 1**).

Of the 10,330 patients included (median age 34 years-old, 78% male), 1955 (18.9%) had alcohol exposure before road trauma, 2415 (23.4%) had severe trauma, 1360 (13.2%) required ICU admission, and 267 (2.6%) died. Of the 9904 patients (96%) who held a driver’s licence before their first index trauma admission, the average time between first obtaining a driver’s licence and the index trauma admission was 16 years (median 12 years, interquartile range [IQR] 5–23, min-max 0.02–81). Prior traffic offences were recorded in 6269 (60.7%) injured patients; and the median time between the last traffic offence and the road trauma admission was 11 months (IQR 4–28). Motor vehicle drivers (33.8%) and motorcycle riders (35.3%) accounted for about 69% of all road trauma patients during the study period. A history of prior traffic offences was overrepresented among the male patients, motor vehicle drivers, motorcycle riders, those with alcohol exposure prior to the road trauma, those with severe trauma (ISS >15), head or spinal column trauma, and those who required ICU admission or died. Indeed, no death was observed among those without prior traffic offences (**Table 1**).

**Table 1:** Differences in characteristics and outcomes between those with and without prior traffic offences (N = 10,330).

**Variables**	**All patients (N = 10,330)**	**Patients with prior traffic offences (n = 6269)**	**Patients without prior traffic offences (n = 4061)**	**P value** ^#^
Age, years (Q1-4)	34 (23–51)	34 (23–50)	34 (22–53)	0.513
Male, no. (%)	8002 (77.5)	5024 (80.1)	2978 (73.3)	0.001
Role in the accident, no. (%):				
Motor vehicle driver	3495 (33.8)	2234 (35.7)	1261 (31.1)	0.001
Front seat passenger	892 (8.6)	465 (7.4)	427 (10.5)	0.515
Back seat passenger	424 (4.1)	221 (3.5)	203 (5.0)	0.043
Motorcycle rider	3642 (35.3)	2362 (37.7)	1280 (31.5)	0.001
Motorcycle pillion	120 (1.2)	76 (1.2)	44 (1.1)	0.683
Pedal cyclist	1067 (10.3)	541 (8.6)	526 (12.9)	0.001
Pedestrian	690 (6.7)	370 (5.9)	320 (7.9)	0.001
Alcohol exposure prior to the accident, no. (%)	1955 (18.9)	1320 (21.1)	635 (15.6)	0.001
Duration of having traffic licence prior to accident, years (Q1-4)*	12.3 (3.8–26.7)	12.3 (4–26.6)	12.4 (3.3–27.1)	0.219
Injury Severity Score [ISS] (Q1-4)	9 (4–17)	10 (4–22)	6 (4–10)	0.001
Severe trauma (ISS>15) (%)	2415 (23.4)	2287 (36.5)	128 (3.2)	0.001
Head and/or spinal column injury, no. (%)	3164 (43.8)	3164 (50.5)	1359 (33.5)	0.001
Required ICU admission (%)	1360 (13.2)	1210 (19.3)	150 (3.7)	0.001
Length of ICU stay, days (Q1-4)**	5 (2–12)	4 (2–12)	6 (2–13.8)	0.124
Length of hospital stay, days (Q1-4)	4 (2–12)	5 (2–14)	3 (2–8)	0.001
Hospital mortality, no. (%)	267 (2.6)	267 (4.3)	0 (0)	0.001

Note: All continuous data are described in median with quintiles (Q1-4, 20th and 80th percentile). ICU, intensive care unit.

^#^P values generated by Chi square or Mann-Whitney test.

*426 individuals did not have driver’s licence prior to admission related to motor vehicle trauma.

**Analysis included only those admitted to the ICU (n = 1360); length of ICU stay was significantly longer among those with prior traffic offences if all patients were included in this analysis.

### Primary Outcomes

The cumulative number of prior traffic offences (odds ratio [OR] per offence 1.03, 95% confidence interval [CI] 1.02–1.05) or demerit points (OR per demerit point 1.02, 95%CI 1.01–1.03) was significantly associated with an increased risk of alcohol exposure prior to road trauma, after adjusting for age, gender and duration of holding a driver’s licence prior to the road trauma. Similar associations between severe trauma (ISS>15) and the cumulative number of prior traffic offences (OR per offence 1.14, 95%CI 1.13–1.15) or demerit points (OR per demerit point 1.05, 95%CI 1.04–1.06) were also observed. In terms of risk of ICU admission or death after road trauma, almost exactly the same patterns were observed (OR per offence 1.10, 95%CI 1.08–1.11; OR per demerit point 1.08, 95%CI 1.07–1.09).

Drink-driving (OR 2.83, 95%CI 2.22–3.61), seat-belt (OR 1.64, 95%CI 1.38–1.94), and use of handheld electronic device offences (OR 1.40, 95%CI 1.02–1.92) were specific offences strongly associated with road trauma leading to ICU admission or death—all in a ‘dose-related’ fashion—after adjusting for age, gender and duration of holding a driver’s licence (**Table 2**). For example, a patient who had accumulated three drink-driving offences had a risk of >80% in having subsequent severe trauma requiring ICU admission or death compared to only a risk of 3.7% for another patient who did not have any prior traffic offences.

**Table 2:** Relationships between cumulative number of prior traffic offences, demerit points, or specific types of traffic offences of a licenced motorcycle rider or motor vehicle driver and risk of road trauma leading to intensive care admission or death (n = 1020, N = 7137).

**Variables**	**Odds ratio (95% confidence interval)**	**P value**
**Model 1:**		
Age, *per 10 years increment*	1.02 (1.01–1.03)	0.001
Male	1.18 (0.98–1.42)	0.081
Duration of holding a driver’s licence, *per year increment*	0.99 (0.98–0.99)	0.036
Number of prior traffic offences, *per traffic offence increment*	1.10 (1.08–1.11)	0.001
**Model 2:**		
Age, *per 10 years increment*	1.02 (1.01–1.03)	0.001
Male	1.06 (0.87–1.28)	0.567
Duration of holding a driver’s licence, *per year increment*	0.99 (0.98–0.99)	0.021
Number of prior demerit points, *per demerit point increment*	1.08 (1.07–1.09)	0.001
**Model 3:**		
Age, *per 10 years increment*	1.03(1.02–1.04)	0.001
Male	1.21 (0.99–1.47)	0.054
Duration of holding a driver’s licence, *per year increment*	0.99 (0.98–0.99)	0.041
Number of specific prior traffic offences: *odds ratio per specific traffic offence*		
Over-speeding	1.01 (0.99–1.04)	0.308
Drink-drinking	2.83 (2.22–3.61)	0.001
Seatbelt	1.64 (1.38–1.94)	0.001
Hoon law	1.26 (0.89–1.80)	0.195
Red traffic light	0.92 (0.79–1.06)	0.238
Use of handheld electronic devices	1.40 (1.02–1.92)	0.038
Careless driving	1.42 (0.99–2.04)	0.058
Bicycle related	1.12 (0.90–1.38)	0.307
Others	0.99 (0.90–1.09)	0.834

Note: The proportion of patients correctly classified by model 1, 2, and 3 are 85.4%, 84.4%, and 85.4%, respectively.

**Fig 2:**
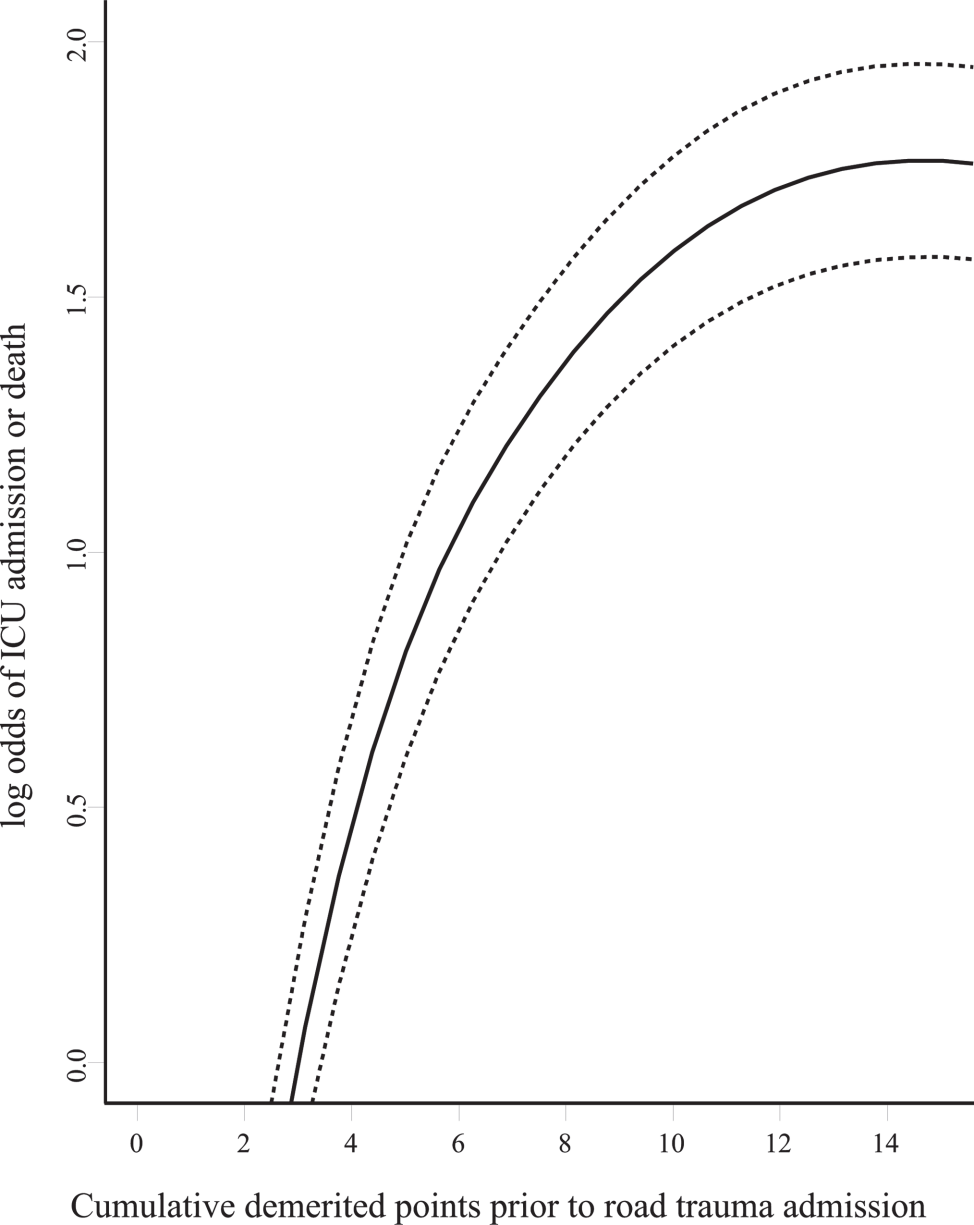
Relationship between cumulative traffic offences demerit points and risk of requiring intensive care unit (ICU) admission or death from road trauma. Dotted lines delineate 95% confidence interval.

In the sensitivity analysis, we confirmed that the risk of subsequent severe trauma was related to cumulated demerit points in a relatively linear fashion (**Fig 2**).

### Secondary outcomes

Of the 10,063 road trauma patients who survived their first road trauma admission, a total of 160 patients were readmitted to the State Trauma Centre due to road trauma subsequently during the study period. The number of traffic offences (OR 1.04, 95%CI 1.01–1.07) or demerit points (OR 1.02, 95%CI 1.01–1.03) *subsequent* to their first road trauma admission were associated with an increased risk of readmission due to road trauma. Seat-belt offences subsequent to the initial trauma admission were the only specific traffic offences associated with an increased risk of readmission due to road trauma (**Table 3**). The average follow-up time between index trauma admission and the censored date (June 2014) for subsequent traffic offences was 6.9 years (median 6 years, IQR 3–10, min-max 0.4–16).

**Table 3:** Factors associated with readmissions to the State Trauma Centre from injury after road trauma among those who recovered from their first injury during the study period (n = 160, N = 10,063).

**Variables**	**Odds ratio (95% confidence interval)**	**P value**
**Model 1:**		
Age, *per 10 years increment*	0.98 (0.95–0.99)	0.026
Male	1.64 (1.03–2.61)	0.038
Duration of holding a driver’s licence before the first injury, *per year increment*	1.01 (0.99–1.04)	0.409
Number of traffic offences *after* the first injury, *per traffic offence increment*	1.04 (1.01–1.07)	0.011
**Model 2:**		
Age, *per 10 years increment*	0.97 (0.95–0.99)	0.024
Male	1.64 (1.03–2.61)	0.038
Duration of holding a driver’s licence before the first injury, *per year increment*	1.01 (0.99–1.04)	0.399
Number of demerit point *after* the first injury, *per demerit point increment*	1.02 (1.01–1.03)	0.012
**Model 3:**		
Age, *per 10 years increment*	0.98 (0.95–0.99)	0.027
Male	1.64 (1.03–2.62)	0.038
Duration of holding a driver’s licence before the first injury, *per year increment*	1.01 (0.99–1.04)	0.332
Number of specific traffic offences *after* the first injury, *odds ratio per specific traffic offence*		
Over-speeding	1.02 (0.98–1.06)	0.390
Drink-drinking	1.65 (0.97–2.80)	0.063
Seatbelt	1.44 (1.06–1.95)	0.021
Hoon law	0.82 (0.28–2.43)	0.720
Red traffic light	1.12 (0.83–1.52)	0.460
Use of handheld electronic devices	0.66 (0.35–1.24)	0.194
Careless driving	1.17 (0.61–2.26)	0.640
Bicycle related	1.25 (0.70–2.22)	0.449
Others	1.15 (0.92–1.44)	0.228

Note: The proportion of patients correctly classified by model 1, 2, and 3 are all 98.4%.

**Fig 3:**
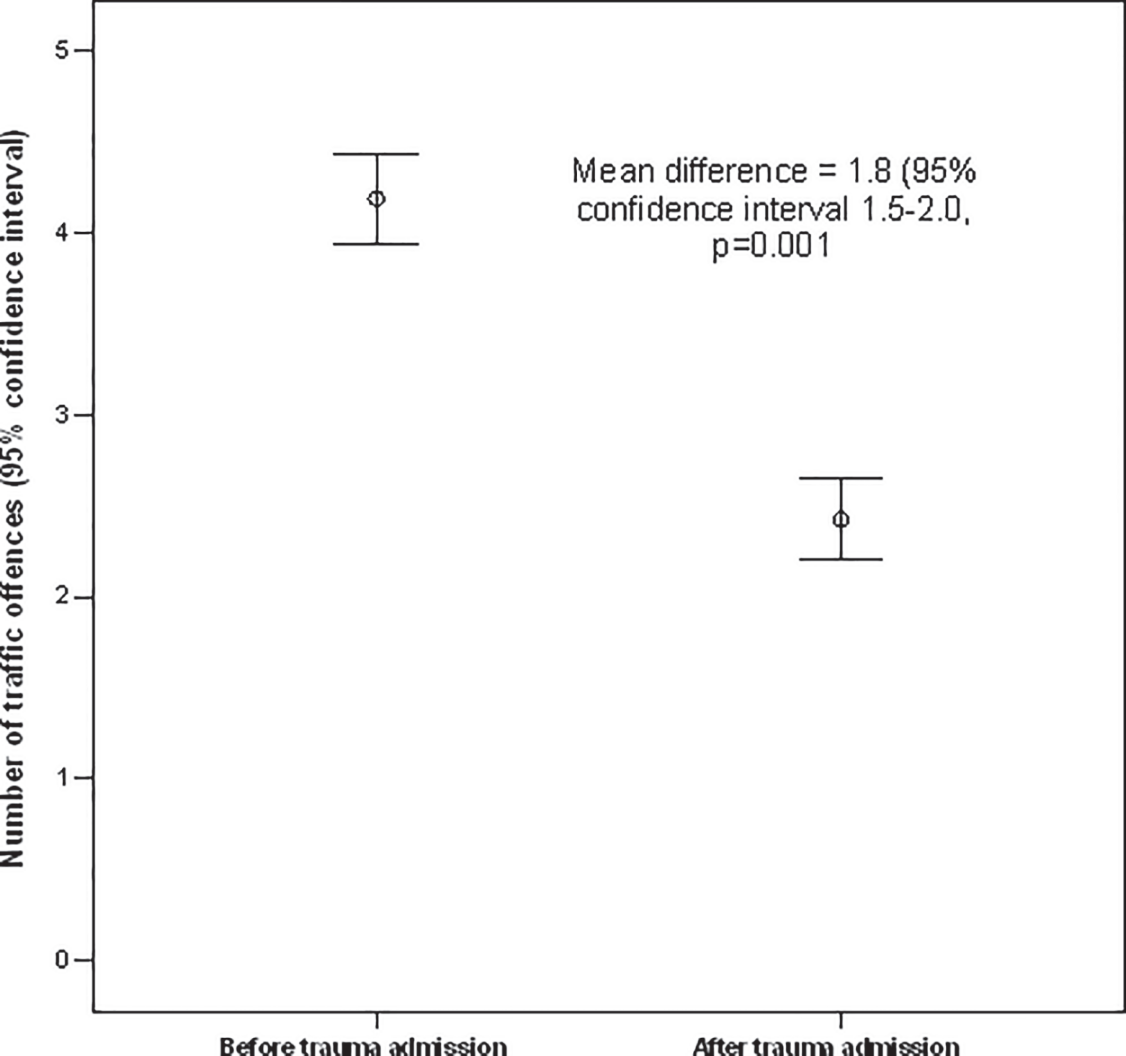
Differences in number of traffic offences between before and after road trauma requiring intensive care unit admission. These results remained unchanged (reduction in number of traffic offences = 1.6, 95% confidence interval 1.1–2.0, p = 0.001) after including only those with at least one traffic offence after their injury.

**Fig 4:**
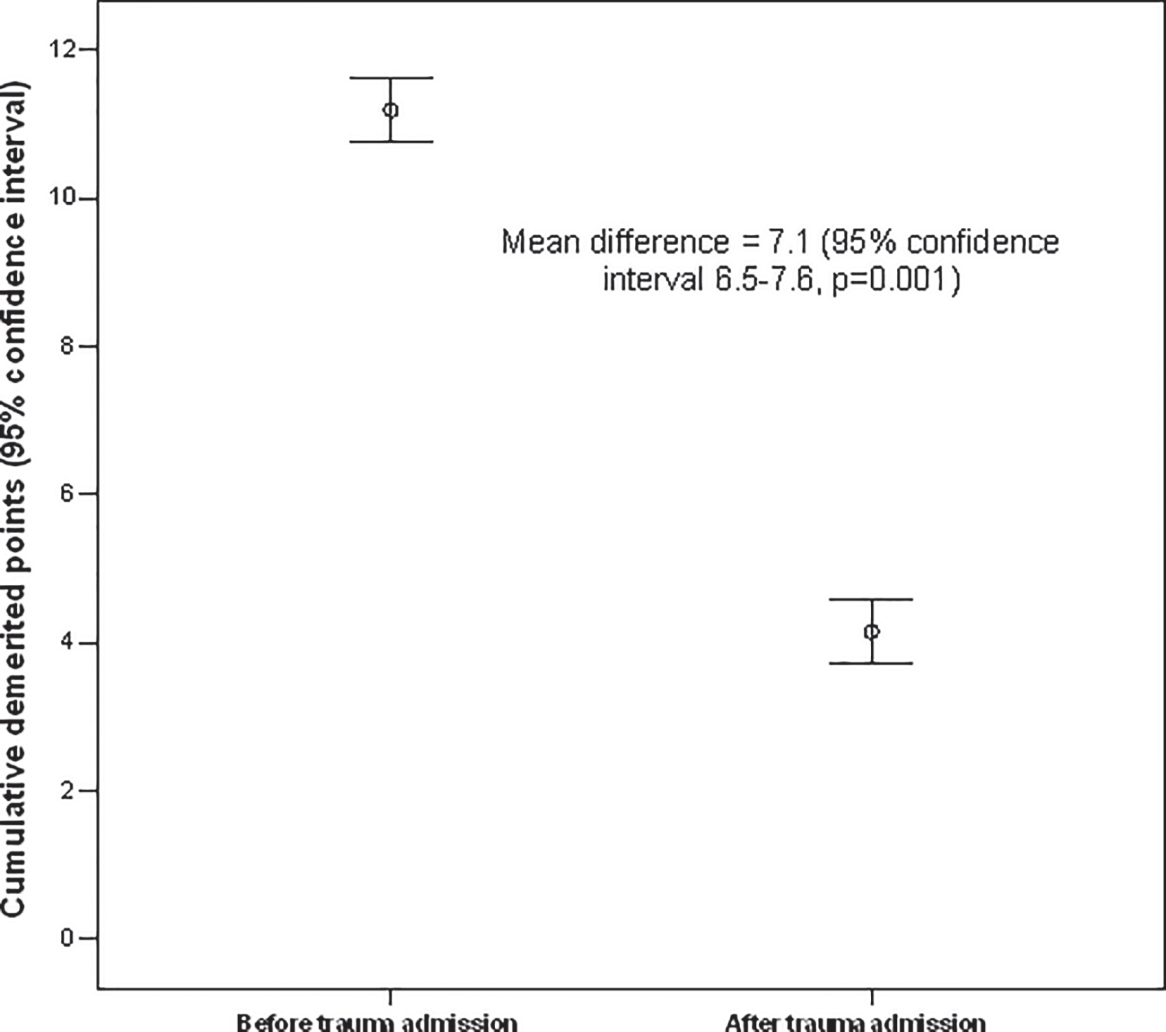
Differences in number of demerit points between before and after road trauma requiring intensive care unit admission. This result remained unchanged (reduction in number of demerit points = 3.7, 95% confidence interval 2.9–4.5, p = 0.001) after including only those with at least one traffic offence after their injury.

When we examined patients who recovered from severe road trauma requiring an ICU admission, there was a suggestion that these patients did, on average, have a lower incidence of *subsequent* traffic offences (absolute mean difference 1.8, 95%CI 1.5 to 2.0) or demerit points (absolute mean difference 7.0, 95%CI 6.5 to 7.6) compared to before their first road trauma admission (**Figs 3 and 4**). This result remained unchanged by comparing the number of traffic offences or demerit points per year of follow-up between before and after the injury. Restricting this latter analysis only to those who had committed at least one traffic offence subsequent to their first road trauma did not change the results.

## Discussion

This study shows that prior traffic offences were common in patients involved in road trauma, and they were associated with alcohol exposure prior to road trauma and severe injury requiring ICU admission or death. The relationship was clearly dose-related and proportional to the number of demerit points and the number of traffic offences. The nature of the traffic offences was also important. Patients who recovered from severe injury requiring ICU admission appear to have reduced their subsequent traffic offences, but those who re-offended were more likely to be readmitted again due to road trauma.

These findings have strong public health significance and require several areas of further discussion. First, we established that traffic offences were common in patients involved in road trauma (60.7%), a proportion that was higher than all licenced drivers with traffic offences (51.3%, mean difference 9.4%, 95%CI 8.5–10.4) in the state. It appears that many road trauma injuries did not occur merely by chance; instead what we observed was a pattern of risk-taking behaviours and the associated consequences [9]. Prior traffic offences were related to an increased risk of alcohol exposure before road trauma and also severe trauma or death. Given the median time between the last traffic offence and the road trauma admission was relatively long (11 months), we argue that there is a window of opportunity to intervene and reduce their risk of severe road trauma. This window of opportunity should be used to trial and evaluate injury prevention education programs, health promotion programs, the effectiveness of suspending drivers’ licences at a lower level of cumulative demerit points, criminalizing traffic offences [10], or other innovative interventions. A previous study showed that juvenile justice offenders with a history of traffic or violence related offences had a very high risk of subsequent re-offending (27%), injury requiring hospitalisation (1.6%) or death, unless active intervention in the form of injury awareness education was provided [3]. Because every road trauma is costly in both human and financial terms (A$22,217 per serious injury) [11], more action is definitely needed to protect all road users including the cyclists and pedestrians. Even simple injury awareness education program can be highly cost-effective compared to many existing medical therapies [11].

Second, for those who recovered from their severe injury requiring ICU admission and resumed driving, they had a lower incidence of traffic offences compared to before their injury (**Figs 3 and 4**). It is possible that a near death experience after road trauma may have helped some of them to realise how close they were to dying on the roads. In fact, the Canadian ‘Prevent Alcohol and Risk-related Trauma in Youth (P.A.R.T.Y.)’ injury awareness education program has a major component allowing the participants to experience how road trauma can result in critically injured patients in an ICU [3,12]. Nevertheless, our study also shows that not all road trauma patients became perfect drivers—a small proportion of them remained resistant to change resulting in subsequent hospital readmissions or even deaths from road trauma [13]. Young male drivers again appear to be those who are most difficult to change (**Table 3**) [4]. Perhaps, intensive education interventions need to be targeted to those who are particularly at risk of having further traffic offences leading to further road trauma [14].

Third, although many countries have experienced decreases in road traffic fatalities since 2007, many middle-income countries have also experienced an increasing number of road trauma fatalities (>20.1 per 100,000 population) compared to high-income countries (8.7 per 100,000 population) and low-income countries (18.3 per 100,000 population) [15]. In addition to policies to enforce safe speed limit, no drink-driving, use of helmets, seat-belts, and child restraints, perhaps intensive road safety education interventions should also be seriously considered for many millions of new motor vehicle drivers in the middle-income countries.

Our study has some limitations. First, the strength of the relationship between severe road trauma and prior traffic offences may have been underestimated, as patients with repeat admissions and also traffic offences that were directly linked to road trauma admissions were excluded from our primary outcome analyses. Second, motor vehicle fatalities per population (9 per 100,000 per year between 2001 and 2009) in the state of this study are slightly higher than some other states. Hence our results may not be completely applicable to other jurisdictions [16]. Third, although we had data on individuals’ duration of having a driver’s licence and all the possible traffic offences recorded within Department of Transport data system, it is possible that some patients might have incurred traffic offences in other states or even overseas, and these data were not available to us for analysis in this study. Finally, although many health promotion and injury education programs appear promising in reducing participants’ future risk of injury, whether these interventions can be incorporated into the current traffic offences penalty system remains uncertain, but is worth further consideration by government.

In conclusion, prior traffic offences were a significant risk factor for alcohol-related road trauma and severe road trauma leading to ICU admission or death. Patients who recovered from severe injury requiring ICU admission appear to have reduced their subsequent traffic offences, but those who re-offended were more likely to be readmitted again due to road trauma. Monetary fines for traffic offences alone may not be adequate in reducing traffic offenders’ risk of subsequent severe injury leading to ICU admission or death.

## Supporting Information

S1 Text The STROBE checklist. (DOC)
